# The Relationship Among Expectancy Belief, Course Satisfaction, Learning Effectiveness, and Continuance Intention in Online Courses of Vocational-Technical Teachers College Students

**DOI:** 10.3389/fpsyg.2022.904319

**Published:** 2022-06-21

**Authors:** Jian-Hong Ye, Yi-Sang Lee, Zhen He

**Affiliations:** ^1^Faculty of Education, Beijing Normal University, Beijing, China; ^2^Department of Industrial Education, National Taiwan Normal University, Taipei, Taiwan

**Keywords:** COVID-19, classes suspended but learning continues, expectation confirmation theory, online course, vocational-technical teacher education

## Abstract

Since the beginning of year 2020, when the whole world were undergoing the COVID-19 epidemic, all schools were lockout and classes were suspended until e-learning was rushed to be online for students to continue their learning, including the students in China. Although many studies had discussed the effectiveness of online learning from many different points of views, it still remained many uncertainties on the qualities of distance learning, especially when under the circumstances of rush and students’ involuntary learning. This manuscript attempted to determine whether students’ learning expectation reach the qualities of distance learning or not. In this manuscript, the snowball sampling method was adopted to have 356 students who studied at vocational-technical teachers’ college in China. Based on the expectation confirmation theory and its model, five hypotheses were proposed to construct a research model to determine relationship between student’s expectancy belief, course satisfaction, learning effectiveness, and continuous learning intention when facing the dilemma of classes suspended but learning continues, learning online during this ongoing pandemic. The results of this study showed that: (1) The expectancy value belief were positively related with theoretical course satisfaction, but negatively related with practical course satisfaction; (2) Theoretical course satisfaction and practical course satisfaction were positively related with learning effectiveness; and (3) Learning effectiveness was positively related with continuance to learn. In addition, three factors influencing the most on the qualities of theoretical course were environmental interference such as surrounding noises, poor internet connection, and poor absorption of learning contents, respectively, while three factors influencing the most on the qualities of practical course were inaccessible to practice, poor absorption of learning content, and lack of practical materials, respectively. Based on the results analyzed, this study suggested schools and teachers focused on how to improve the qualities and to reduce or prevent any disturbances to the class given to fulfill students’ class expectation first and then to ensure students’ learning effectiveness and intention to continuous learning.

## Introduction

Due to the large-scale outbreak of COVID-19 starting from year 2020, colleges and universities around the world were facing unprecedented challenges, including many schools closing their campuses and suspending physical teaching and learning activities (Chen et al., 2021). Nevertheless, for the countries with complete network infrastructure setup and online learning administration system, immediate implementation of digital distance education played a pivotal role (Müller et al., 2021). To many researchers and online teachers, online learning during the pandemic helped to address the shortcomings of traditional face-to-face education (Wang T. et al., 2021).

In February 2020, the Ministry of Education of China issued the “Guiding Opinions on Doing a Good Job in the Organization and Management of Online Teaching in Ordinary Colleges and Universities during the Period of Epidemic Prevention and Control,” requiring all colleges and universities to actively conduct online teaching and learning or other non-face-to-face teaching activities. As a result, most online courses in colleges and universities in China were conducted with traditional teacher-centered lectures, and students learned by viewing the lectures through electronics consumer products during the COVID-19 lockdown (Hong et al., 2021). Although distance teaching has been promoted and implemented for many years before the outbreak of COVID-19, most of the early research showed that distance teaching was conducted with the consents and fully preparations of teachers and students in advance, and students were able to choose other learning method freely as well. Unlike other studies, this study collected its research data in the circumstance of COVID-19 epidemic, when colleges and universities only opened for a limited time and students were unable to return to schools in person and somehow forced into distance teaching under pressure (Lo et al., 2021). To many educational institutions, this transitional approach was temporarily carried out on an *ad hoc* basis for a short period of time, so there was little planning or experience to guide out how to conduct distance courses (Orlov et al., 2021). The rapid change and unexpectancy enforcement from face-to-face teaching to distance learning also highlighted many challenges and constraints (Carrillo and Flores, 2020). Despite of many advantages and benefits of implementing online courses during the COVID-19 epidemic, the satisfaction and intention of students for online courses was still unknown. This study tried to help teachers to design more suitable online courses to meet students’ expectations in the future.

Based on the viewpoints of marketing theory, Expectation Confirmation Theory (ECT) proposes that consumers form expectations (expectancy beliefs) for a product or service before purchasing a product or service, and they will confirm the degree of self-expectation (satisfaction) to the product or service after actual purchase and consume (Oliver, 1980; Lin et al., 2005). In other words, when students take online courses at the beginning or before starting the online courses, they will have expectancy beliefs regarding to this teaching approach, and of course they will perceive degree of satisfaction to online courses after actually experiencing the online courses.

In addition, Bhattacherjee (2001b) proposed an Expectation Confirmation Model (ECM) based on ECT to assess the level of personal satisfaction and expectation, and also to make more predictions about the model and its exploration, which can be understood better about the persistence of the model usage (Tam et al., 2020), since consumers’ overall satisfaction constitutes their post-purchase intentions (Hossain and Quaddus, 2012). In sum, after knowing the satisfaction of students, it is necessary to study the students’ intention to continue on-line learning after taking online courses in order to have a more comprehensive understanding of students’ usage of online courses.

Expectation Confirmation Model originated from market research, which was based on difference theory to explain the formation of satisfaction that was the relationship between initial expectation and satisfaction derived from difference theory (Dai et al., 2020). ECT and ECM have been widely applied to survey consumers’ post-use behavior (Bhattacherjee, 2001b), such as predicting students’ continuing reading of e-books (Stone and Baker-Eveleth, 2013), learning management systems (Cheng and Yuen, 2018), and Chinese university MOOCs (Dai et al., 2020) which is a mature theoretical model that can be used to explore the satisfaction of online course learners. Based on the above mentioned, this study will use ECT and ECM as the theoretical basis of this study.

Since the outbreak of the pandemic, there were many studies discussing all kinds of issues regarding online learning for different learning groups, such as medical and dental students’ satisfaction and willingness to use online learning (Rajeh et al., 2021), college students’ satisfaction and usage retention rate on digital learning platforms (Salimon et al., 2021), technical college students’ satisfaction with blended learning programs (Huang, 2021), college students’ satisfaction with flipped learning (Kim et al., 2021), and continued willingness for different video learning formats (Wang et al., 2022), etc.

However, only a brief understanding regarding college students’ satisfaction and continuance intentions to study online during the period of the pandemic was revealed; more studies were still investigating other topics such as: online learning satisfaction of the students who took different online learning courses such as theoretical courses and practical courses at the same time, disturbing factors intervening the quality of these two different styles of courses, and online learning satisfaction of vocational and technical normal students, etc., which were also rarely discussed. Nevertheless, it was important and meaningful to explore student satisfaction degree with online learning (Jiang et al., 2021). Therefore, in order to extend the understanding of this topic, the purpose of this study was to explore, during the COVID-19 period, the relationships between the expectancy beliefs of about adopting online courses, the satisfaction of two different courses (theory courses and practical courses), learning effectiveness and intention of continuous e-learning of college students in vocational and technical training courses.

## Literature Review

### Vocational and Technical Teachers Education

Vocational and technical teachers in China are mainly trained by vocational and technical normal universities (Wu and Ye, 2018). After the implementation of the “Teachers Law” and the “Vocational Education Law” in the 1990s, vocational and technical education and teaching institutions were established and developed in China (i.e., vocational and technical teacher training institutions), also known as higher vocational and technical teacher colleges (Billett, 2009). Higher vocational and technical teacher education is mainly to cultivate professional teachers needed for vocational education (Sun, 2009). In China, vocational teachers can be sorted into two groups: cultural course teachers and professional course teachers. Cultural course teachers mainly teach general knowledge courses such as Chinese, English, mathematics, and history, while professional course teachers are sub-sorted into theoretical course teachers and practical course teachers (Billett, 2009). A professional course teacher should have the knowledge, skills, and ability related to teaching subjects. More than 160 universities in China have established vocational and technical normal colleges or become vocational and technical normal universities. The number of students in this field is around 21,000 (Wu and Ye, 2018). In this study, the vocational and technical normal students referred to the college students who studied professional subjects in higher vocational and technical normal colleges. At present, due to the pandemic, the research on vocational and technical normal students was almost down to zero, but the teacher training in vocational education was the keystone to cultivate the vocational education system. Therefore, it was necessary to understand the online learning situation of vocational and technical students during this special period. As a result, this study used vocational and technical students as participants to explore their perceived outcomes of taking online courses during the COVID-19 pandemic.

### Expectancy Beliefs for Online Courses

Bandura (1977) defined outcome anticipatory beliefs as a person’s assessment that a particular action delivered certain outcomes. It is a belief in a desired outcome which means that people believe that a particular action lead the expected result (Yesilyurt et al., 2021; Davis et al., 2022). Therefore, when people evaluate the results with higher expectations, it reflects the higher expectations for effectiveness (Molloy and Anderson, 2022). In addition, expectancy beliefs are also defined as the degree to which a person believes that a system can help them improve their performance (Chiu and Wang, 2008). In the field of education, expectancy beliefs are understood as anticipatory predictions made by students themselves trying to anticipate their own behavior, emotions, and outcomes in a new educational environment (Doménech-Betoret et al., 2017). Eccles and Wigfield (2002) defined positive anticipatory beliefs were the beliefs about an individual’s ability to complete different learning tasks in a specific area, either immediately or in the future. Research indicates generating positive anticipatory beliefs of committing to use online technology will lead to effective learning, and such learning will lead to outcomes valued critically by students as students’ commitment to use online learning systems (Bates and Khasawneh, 2007). It represents students’ belief that participation in online activities will promote desirable learning outcomes and the perceptions of outcomes and achievements will motivate individuals to use the technology for beneficial outcomes (Yang et al., 2007). In sum, this study takes expectancy beliefs as a factor to discuss participants’ expectations of on-line learning performance during the pandemic.

### Course Satisfaction for Online Courses

According to Palacio et al. (2002), satisfaction occurs when perceived performance meets or exceeds individual expectations. Satisfaction refers to the post-assessment of a consumer’s initial (trial) experience with a service, expressed as positive feelings (satisfaction) or indifferent or negative feelings (dissatisfaction) (Bhattacherjee, 2001a; Chen and Lin, 2019). More specifically, assessments that exceed expectations lead to higher satisfaction, while assessments that fail to meet expectations lead to lower satisfaction (Kujala et al., 2017). Furthermore, satisfaction could be said to deliver the right knowledge to the right person at the right time (Alsharyofi, 2022). In the field of education, learning satisfaction represents one of the key indicators of learning outcomes (Chou and Liu, 2005). How students view their learning will lead to certain outcomes and the importance they attach toward learning (Cheng et al., 2016). Basically students’ satisfaction with the course is an important outcome because it influences the student’s decision to continue or withdraw from the course (Levy, 2007; Howell and Buck, 2012). It also makes student satisfaction an important predictor of success and achievement across all educational indexes (Hoda et al., 2022).

Course satisfaction is described as short-term attitudes that are assessed through students’ educational experience, services, and facilities (Weerasinghe and Fernando, 2007), which means the learning satisfaction with a course may reflect students’ subjective evaluations, both positive and negative, of using the course (Lin et al., 2022). Student satisfaction is considered as one of important factors when determining the quality of online education (Kurucay and Inan, 2017; Bayrak and Altun, 2020). In general, students’ satisfaction with online courses can tell teachers how students evaluate courses based on teachers’ performance and the overall quality of the learning experience (Gunawardena et al., 2010). Therefore, this manuscript used course satisfaction to discuss the perceived satisfaction of participants participating in online theoretical courses and practical courses during the pandemic.

### Learning Performance of Online Courses

Student learning is the most important outcome of education and perceived learning (self-report of learning) is used to evaluate curriculum quality (Kurucay and Inan, 2017). Learning effectiveness is considered as one of the key indicators of learning outcomes (Chou and Liu, 2005). Learning effectiveness is a positive effect of teachers’ desire for students to acquire learning and also a goal that teachers want learners to achieve (Wu, 2018). The criterions of learning effectiveness can be students’ perceived learning level (Willging and Johnson, 2009), which were the comprehensive results of learners’ learning outcomes (Jouhari et al., 2015). In other words, learning effectiveness refers to the changes induced by training and education, and direct learning effects refer to changes in students’ behavior after lecturing (Lo et al., 2021). From a students’ perspectives, learning performance refers to the perceived value of learning by students (Ganesh et al., 2015), so learning outcomes can be developed as the basis for performance assessment to reflect instructional designs and strategies (Wager and Smith, 2003). Hori (2021) pointed out information and communication technology (ICT) was the major trend of learning method throughout the world and kept having strong impact in either learning or career environment in the future. Since then, effective online learning is believed to bring benefits directly or indirectly to students, so online learning effectiveness refers to the improved ability of students through digital media and online learning process (Hongsuchon et al., 2022). However, during the pandemic, the rapid transition to online learning mode may have affected the learning effect, which is worth to be studied (Tsang et al., 2021). If online teachers and course designers want to ensure the effectiveness of online learning, it is important to understand students’ perceptions of the effectiveness or ineffectiveness of online courses (Hong et al., 2021). In sum, this study uses course satisfaction to discuss participants’ perceptions of their learning performance during online classes during the pandemic.

### Intention to Continue Learning Online Courses

More and more researchers emphasize the needs to understand whether or not technology will continue to be adapted in the future (Joo et al., 2018), while the continuous behavior of usage depends on the continuous intention of the users. Online learning can exert its educational effectiveness only when the learners intend to continuing participating in the learning activities (Li et al., 2021). The intention to continuing participating represents the participants’ adherence toward the activities (Sung and Lin, 2021). When an individual develops a positive attitude and overall attachments to things or activities, there will be continuous intention (Tsai et al., 2018). In the field of education, many studies have adapted the ECT framework to discuss students’ continuous intention (Wang T. et al., 2021). Bhattacherjee (2001b) suggested taking continuous intention as the variable to the acceptance of IT. Course satisfaction to participants’ perceptions of intention to continuous learning through online courses in the future will be discussed in this study.

## Research Method

### Research Model

**FIGURE 1:**
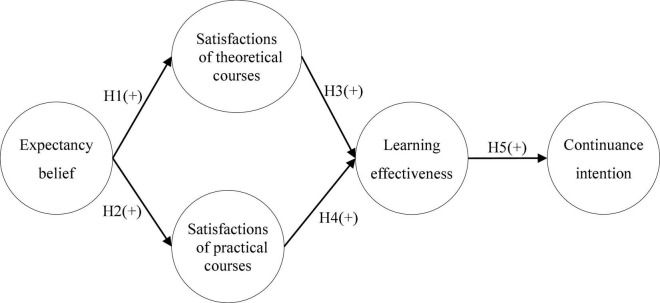
Research model.

Expectation confirmation theory is an important theory to predict and explain the satisfaction and continuous behavior (Wang T. et al., 2021). ECT proposes that users evaluate the perceived performance of a product based on their initial expectations (expectancy beliefs). If a product performs better than expectancy, there may be a positive sense of identity (satisfaction) (Hossain and Quaddus, 2012). Based on the ECM proposed by Bhattacherjee (2001b), he proposed ECM which further extended the relationship between expectations and experience differences (Kujala et al., 2017). To accomplish the goals of this study, this study proposes five hypotheses and constructs a research model to discuss the relationship between expectancy beliefs, course satisfaction, learning effectiveness, and continuance intention based on ECT and ECM, as shown in Figure 1.

### Research Hypothesis

#### Expectancy Beliefs and Course Satisfaction

Expectancy beliefs can be confirmed when perceived performance exceeds expectations. Balanced when perceived performance equals expectations or rejections when perceived performance is lower than expectancy occurs depending on how well users experience, whether they will be satisfied or dissatisfied (Chiu et al., 2005). Therefore, ECT recognizes expectancy performance as an important determinant of predicting satisfaction, because expectancy beliefs provide users with a benchmark or reference level to form judgment criteria for key services (Bhattacherjee, 2001b). People’s perceived level of satisfaction is determined from the initial expectancy beliefs of services and the differences between services (Thong et al., 2006). Bhattacherjee (2001b) proposed that user satisfaction is determined by prior experience and expected usefulness. In other words, when people receive services that meet or exceed expectations, there will be positive satisfaction.

It is found in past researches that expectancy beliefs have a positive impact on students’ perceived satisfaction (Doménech-Betoret et al., 2017). From the perspective of Expectancy value theory, students’ perceived satisfaction will depend on students’ expectancy beliefs prior to actual learning (Cheng et al., 2016). At the end of the course, students’ expectancy beliefs and perceptions in the course were investigated and found that the degree to which students’ expectations were met would be a positive predictor of satisfaction (Appleton-Knapp and Krentler, 2006). Therefore, when students have better satisfaction with online courses, they will also experience higher levels of learning performance. Based on this assumption, this study proposes the following research hypotheses on expectancy beliefs and course satisfaction:

H1: There is a positive relationship between expectancy beliefs and theoretical course satisfaction.
H2: There is a positive relationship between expectancy beliefs and practical course satisfaction.

#### Course Satisfaction and Learning Effectiveness

Expectation confirmation theory proposes a tight relationship between satisfaction and performance confirmation (Dai et al., 2020). When a product or service has good usability, it means it is easy to use and useful to users (Kujala et al., 2017). It was also confirmed that the easier the students’ usage of chosen interfaces, more positive effects on students’ perceived usefulness that also had a direct effect on students’ intentions to continue online learning (Hariguna, 2021). In other words, when students are satisfied with the course, the learning effectiveness brought by the course should be more positive. In educational research, satisfaction has been widely used as an indicator of learning effectiveness (Alavi et al., 1995).

Usually, learning satisfaction is a key indicator of learning effectiveness, so learning satisfaction, as a subjective factor, has a certain impact on learning effectiveness (Li and Liang, 2020). Furthermore, research also indicates that student satisfaction is an important factor to consider when developing a curriculum, as it is directly related to student learning and success (Wickersham and McGee, 2008). Learner satisfaction is an important factor affecting the effectiveness of online courses (Willging and Johnson, 2009). When students have better satisfaction with online courses, they will feel the higher level of satisfaction as well as learning effectiveness. As the world was driving quickly to new technology, things were changed gradually from “the real world” to “the virtual world” (Maillard, 2021), all teachers and students must be able to adapt online class as routine. Accordingly, this study proposes the following research hypotheses on course satisfaction and learning effectiveness:

H3: There is a positive relationship between theoretical course satisfaction and learning effectiveness.
H4: There is a positive relationship between practical course satisfaction and learning effectiveness.

#### Learning Effectiveness and Continuance Intention

Expectation confirmation theory provides a good basis for explaining the relationship between satisfaction and continuance intention (Wang T. et al., 2021). Studies indicate that when an individual’s level of affirmation about perceived performance is critical in explaining continuance intention (Dai et al., 2020), specifically, when users find a product that helps to improve efficiency and they are more willing to continue using it (Kujala et al., 2017). The rationale for the effect of learning behavior on continuance intention is that the more users understand the benefits of using the tool, the more likely they will want to continue using the tool (Bai et al., 2021).

Researches in the past also indicated that for learners with experience in using online learning, the factors that affected their intention to use were perceived benefits and verification results. As a result, perceived positive effectiveness was one of the important factors affecting users’ continuance intention (Hsu et al., 2018). In sum, when students have better satisfaction with online courses, they will also feel a higher level of learning effectiveness. Based on this, this study proposes the following research hypothesis on learning effectiveness and continuance intention:

H5: There is a positive relationship between learning effectiveness and continuance intention.

### Procedure

The snowball sampling method was adopted in this study and the Questionnaire Star platform was used to distribute online questionnaires and research posts (including research purposes, data collection and processing methods, and participant anonymity protection), questionnaire links and QR codes to the social communities. It was filled out by community members of the student exchange community of the vocational and technical normal colleges on group media, such as Weibo and WeChat.

It was assumed that as many as vocational and technical normal students could view this questionnaire through the link as long as they used WeChat or other social media tools. When the participants completed the survey anonymously, they were encouraged to forward the questionnaire link to other friends they knew. The questionnaire was collected from June 15, 2020 to June 30, 2020, and the questionnaires were distributed to Undergraduate students who were the higher vocational and technical students of the Teachers College in China.

### Participants

The number of participants in this study (the number of questionnaires returned) was 419 with a total of 63 invalid data, so the number of valid research participants was 356; the effective data rate was 85%. The average number of hours of online courses per week was 12.91 h (standard deviation 3.21 h); the average age of the participants was 20.51 years (standard deviation 1.54 years). The background information of other participants was shown in Table 1.

**TABLE 1:** Background information of participants.

Categories	Contents	
Gender	Male: 205 (57.6%)	Female: 151 (42.4%)
Grade	First grade: 41 (11.5%)	Sophomore: 154 (43.3%)
	Junior grade: 109 (30.6%)	Senior: 52 (14.6%)
Studying professional fields	Hospitality and tourism: 10 (2.8%)	Architecture: 49 (13.8%)
	Mechanical: 73 (20.5%)	Mechatronics: 63 (17.7%)
	Electronic electrician: 22 (6.2%)	Computer: 22 (6.2%)
	Chemical: 8 (2.2%)	Agriculture: 7 (2.0%)
	Accounting: 44 (12.4%)	Marketing: 20 (5.6%)
	Arts: 11 (3.1%)	Physical education: 4 (1.1%)
	Hair and image: 1 (0.3%)	Video and film: 8 (2.2%)
	Clothing: 3 (0.8%)	Broadcasting and program hosting: 4 (1.1%)
	Dance performance: 2 (0.6%)	Preschool education: 5 (1.4%)
Major online course Tools (Choose 3 the most)	MOOCs: 30 (28.7%)	WeChat: 257 (24.1%)
	Tencent course: 217 (20.3%)	School online: 131 (12.3%)
	DingDing: 62 (5.8%)	QQ live: 46 (4.3%)
	Zoom: 19 (1.8%)	Others: 30 (2.7%)
Online before the pandemics	Experienced: 336 (94.4%)	Non-experienced: 20 (5.6%)
Types of online course	Theoretical courses: 93 (26.1%)	Skills courses: 32 (4.5%)
	Fine Arts courses: 227 (63.8%)	None: 20 (5.6%)

### Questionnaire

The questionnaire used in this study was developed and revised from past research and related theories. The content of the questionnaire was reviewed by two scholars in the field of teacher training and one scholar in digital learning. The review was focused on the text description, the fluency and comprehension of the text, as well as the completeness of the aspect connotation of each question on the questionnaire for three times; 15 Chinese vocational and technical teachers were invited to fill in the testing questionnaire, and then the text content was slightly revised according to the feedback lastly. The content of the questionnaire was evaluated on a 5-point Likert scale: 1 = representing strongly disagree, 2 = representing disagree, 3 = representing average, 4 = representing agree, and 5 = representing strongly agree. To acquire a deeper understanding of the external factors that affect the quality of online learning, this study also designed another questionnaire for students to check for the external factors that affect the learning quality of online courses. One participant could choose three external influences for each theoretical and practical course.

#### Expectancy Beliefs

In this study, expectancy value refers to learners’ perceptions that their learning expectations can be matched with the goals of online learning. Therefore, according to this definition, this study modified the Bates and Khasawneh (2007) scale to measure participants’ belief perceptions about the expectancy value of online course learning, with a total of eight questions. For example: I expect to learn more professional knowledge required for future teaching through online courses. The Cronbach’ alpha of the original scale was 0.87.

#### Course Satisfaction

In this study, course satisfaction refers to learners’ perceptions of their learning feedbacks through online learning. Therefore, according to this definition, this study refers to and modifies the learning satisfaction scale of Wang C.-M. et al. (2021) to measure participants’ satisfaction with online learning theoretical courses and practical courses. There were each six questions for theoretical courses and practical courses with a total of 12 questions. A sample question regarding theoretical course satisfaction was: the online theoretical courses allowed me to learn more about the relevant knowledge needed for future teaching. Another sample question regarding practical course satisfaction was: the online practical course of technical implementation allowed me to learn the professional skills needed for future teaching. The original scale had a Cronbach’ alpha of 0.85, a composite reliability (CR) value of 0.90, an average variance extracted (AVE) value of 0.68, and a factor loading (FL) value of 0.68 (which was between 0.72 and 0.84).

#### Learning Effectiveness

In this study, learning effectiveness refers to learners’ perceptions of meeting their learning expectations through the use of online learning. Therefore, according to this definition, the questions were referred to and modified from the Liaw (2008) e-learning performance scale to measure participants’ perceptions of learning through online courses. The learners’ perception of online learning effectiveness was brought by a total of eight questions. For example: my learning efficiency had been improved since I started online learning. The original scale had a Cronbach’ alpha of 0.72, and factor loading value with a range between 0.75 and 0.76.

#### Continuance Learning Intention

In this study, the continuance learning intention referred to learners’ perceptions of intention the usage of online learning continuously. Based on this definition, refers to and modifies the scale of the continuance intention in this study was referred and modified by the scales designed by Tsai et al. (2018) to measure participants’ perception of intention to continue online learning. There were a total of six questions. For example, I would still take online courses in the future. The original scale had a Cronbach’ alpha of 0.84, a CR of 0.89, an AVE of 0.61, and factor loading value ranged between 0.73 and 0.84.

### Data Analysis

Structural Equation Modeling (SEM) is a powerful and widely used tool, especially in the social sciences. SEM is the first method based on theoretical SEM to define latent variables and then constructs items to measure (Lee Helm et al., in press). Therefore, SEM is the model to help analyzing the measured and structural relationship between variables in a study, i.e., explain how the variables relate to each other (Hansen and Olsson, 2022), which is to evaluate the validity of a theory or hypothesis by using data (Phakiti, 2018). Based on the characteristics of the above statistical methods, this study used the SEM method to verify the research model to test the relationship between expectancy beliefs, two types of course satisfaction, learning effectiveness and continuous intention in online courses. The test criteria and complete verification analysis results were as follows.

## Results

### Item Analysis

In order to confirm the model fit of each aspect used in this study, first-order confirmatory factor analysis was used to conduct item analysis of each aspect to ensure that each aspect had a good degree of fit, and the value of χ^2^/df should be less than 5; RMSEA should be less than 0.10; GFI and AGFI should be higher than 0.80; the convergent validity of the item was confirmed by the factor loading value, and it was recommended to delete when the value was lower than 0.50 (Hair et al., 2010; Kenny et al., 2015). The item analysis resulted of this study were shown in Table 2. The questions of expectancy beliefs were deleted from 8 to 7; the questions of the theoretical course satisfaction was deleted from 6 to 5 items; the questions of the practical course satisfaction was from 6 to 5 items; the questions of learning effectiveness was deleted from 8 to 6 items; continuance intention was deleted from 6 to 5 items.

**TABLE 2:** First-tier confirmatory analysis.

Index	χ^2^	df	χ^2^/df	RMSEA	GFI	AGFI	*t*
Threshold	–	–	<5	<0.10	>0.80	>0.80	>3
Expectancy beliefs	27.10	14	1.94	0.05	0.98	0.96	14.33∼20.20
Theoretical course satisfaction	13.06	5	2.61	0.07	0.99	0.96	13.31∼14.84
Practical course satisfaction	15.84	5	3.17	0.08	0.98	0.95	20.29∼27.70
Learning effectiveness	37.08	9	4.12	0.09	0.97	0.93	13.67∼16.40
Continuance learning intention	19.70	5	3.94	0.09	0.98	0.94	14.01∼17.99

The values of all respondents for each item in this study were divided into the top 27% and the bottom 27% for *t*-test. If the *t*-value was greater than 3, the external validity was considered to be at a significant level. Table 3 showed that the *t*-values of expectancy beliefs ranged between 14.33 and 20.20, the *t*-values of theoretical course satisfaction ranged between 13.31 and 14.84, the *t*-values of practical course satisfaction ranged between 20.29 and 27.70, and the *t*-values of learning effectiveness ranged between 13.67 and 16.40, the *t*-values of continuance learning intention ranged between 14.01 and 17.99, which meant that all items in this study had external validity (Green and Salkind, 2004).

**TABLE 3:** Reliability and validity analysis.

Construct	*M*	*SD*	Skewness	Kurtosis	α	CR	AVE	FL
Threshold	–	–	–	–	>0.70	>0.70	>0.50	>0.50
Expectancy beliefs	3.55	0.59	−0.45	1.59	0.88	0.88	0.51	0.69∼0.76
Theoretical course satisfaction	3.44	0.81	−1.40	2.40	0.91	0.91	0.68	0.79∼0.88
Practical course satisfaction	2.86	0.84	−0.752	−0.40	0.90	0.90	0.64	0.74∼0.83
Learning effectiveness	3.29	0.69	−0.800	1.51	0.87	0.86	0.50	0.65∼0.74
Continuance learning intention	3.32	0.74	−1.00	1.70	0.84	0.84	0.51	0.67∼0.75

### Outer Model Evaluation

Outer model evaluation refers to the relationship between a facet and its observed metrics. When the external model does not have acceptable reliability and validity, the verification of the structural model becomes meaningless (Henseler et al., 2016), therefore, the prerequisites for structural model testing are to confirm the reliability and validity of the external model. Outer model evaluation involves checking the reliability of a single indicator and the reliability of the measurement model for each facet (i.e., internal consistency reliability), and the convergent and discriminant validity of the measure (Hair et al., 2012). The results of the external model evaluation of this study were as follows:

Hair et al. (2010) suggested that if Cronbach’s α was higher than 0.70, it meant that the facet had good internal consistency. Hair et al. (2010) also suggested that the CR value should exceed the standard of 0.70, while the Cronbach’s α of each facet had good internal consistency with the values ranged between 0.84 and 0.91, and CR values ranged between 0.84 and 0.91 that met with the recommended criteria, as shown as in Table 3.

Hair et al. (2010) pointed out that when the FL value of an item was higher than 0.50, the item had convergent validity. The analysis indicated that the FL value of expectancy belief was between 0.69 and 0.76; the FL value of theoretical class satisfaction was between 0.79 and 0.88; the FL value of practical class satisfaction was between 0.74 and 0.83; the FL value of learning effectiveness was between 0.65 and 0.74; the FL value of continuance learning intention was between 0.67 and 0.75, as shown as in Table 2. Hair et al. (2011) suggested that the AVE value must be greater than 0.50 to indicate that the facet had Convergent validity. All AVE values for each dimension in this study were ranged between 0.50 and 0.68, as shown as in Table 3.

The way to confirm the discriminant validity of a facet is to compare the square root value of the AVE of each facet with the correlation between the other facets in the model. When the square root value of the facet’s AVE is higher than the other facets that indicates discriminant validity (Awang, 2015). The analysis results of this study showed that all aspects of the research model had discriminant validity, as shown as in Table 4.

**TABLE 4:** Discriminant validity analysis.

Construct	1	2	3	4	5
1. Expectancy beliefs	(0.71)				
2. Theoretical course satisfaction	0.45	(0.82)			
3. Practical course satisfaction	−0.31	0.06	(0.80)		
4. Learning effectiveness	0.25	0.45	0.24	(0.71)	
5. Continuance learning intention	0.43	0.49	0.08	0.47	(0.71)

*The values on the diagonal were the square root values of AVE, and the other values were the correlation coefficient values.*

### Model Fit Analysis

For SEM analysis, the χ^2^/df value should be less than 5 (Hair et al., 2010), the RMSEA value should be less than 0.10, and the seven indicators such as GFI, AGFI, NFI, NNFI, CFI, IFI, and RFI value should be greater than 0.80 (Abedi et al., 2015), and the binomial values of PNFI and PGFI should be greater than 0.50 (Hair et al., 2010). The SEM analysis results of the model fit test in this study showed that χ^2^ = 594.31, df = 345, χ^2^/df = 1.72, RMSEA = 0.05, GFI = 0.90, AGFI = 0.88, NFI = 0.89, NNFI = 0.95, CFI = 0.95, IFI = 0.95, RFI = 0.88, PNFI = 0.82, and PGFI = 0.76; as a result, it could be concluded that this study model passed the fitness test.

### Path Analysis

**FIGURE 2:**
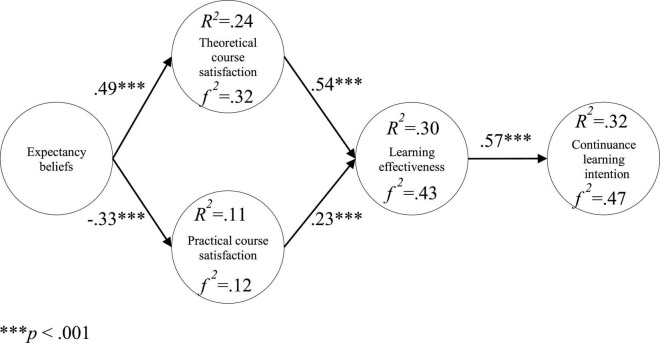
Research model validation. ***p < 0.001.

The model verification showed that: Expectancy beliefs had a positive relationship with theoretical course satisfaction (β = 0.49***; *t* = 7.84); expectancy beliefs had a negative relationship with practical course satisfaction (β = −0.33***; *t* = −5.38); theoretical course satisfaction had a positive relationship with learning effectiveness (β = 0.54***; *t* = 8.72); and practical course satisfaction had a negative relationship with learning effectiveness (β = 0.23***; *t* = 4.25). Learning effectiveness had a positive relationship with continuance learning intention (β = 0.57***; *t* = 8.37), as shown as in Figure 2.

The explanatory power of expectancy beliefs on theoretical course satisfaction was 24%, *f* ^2^ was 0.32; the explanatory power of expectancy beliefs on practical course satisfaction was 11%, *f* ^2^ is 0.12; the explanatory power of two kinds of course satisfaction on learning effectiveness was 30%, *f* ^2^ was 0.43; the explanatory power of learning effectiveness on continuance learning intention was 32%, *f* ^2^ was 0.47, as shown as in Figure 2.

### Indirect Effects Analysis

Indirect effects refer to composite structural paths consisting of two or more direct variable-to-variable paths (Leth-Steensen and Gallitto, 2016). Indirect effect analysis showed that predictive value beliefs had an indirect positive relationship with learning effectiveness and continuance learning intention (β = 0.19*, β = 0.11*); the satisfaction of the two courses also had an indirect positive relationship with continuance learning intention (β = 0.31**, β = 0.13***), and the 95% confidence interval did not include 0 (**p < 0.01), which indicated that two types of course satisfaction played a mediating role between expectancy beliefs and learning effectiveness. The two types of course satisfaction and learning performance played a mediating role between expectancy beliefs and continuous learning intentions, while learning effectiveness played a mediating role between the two types of course satisfaction and continuous learning intentions, as shown as in Table 5.

**TABLE 5:** Indirect effects analysis.

Construct	Expectancy beliefs		Theoretical course satisfaction		Practical course satisfaction	
			
	β	95% CI	β	95% CI	β	95% CI
Learning effectiveness	0.19*	[0.03, 0.34]				
Continuance learning intention	0.11*	[0.01, 0.23]	0.31**	[0.16, 0.49]	0.13***	[0.07, 0.21]

*p < 0.05, **p < 0.01, ***p < 0.001.

### Analysis of External Factors Affecting the Learning Quality of Online Courses

From Table 6, the factors affecting the quality of theoretical courses and practical courses were different. The factors affecting the quality of theoretical courses were ranked as the following: (1) environmental interference factors (such as noise), (2) poor network connection, (3) learning poor content absorption, (4) insufficient interaction between teachers and students, and (5) unsmooth operation of the platform. The factors affecting the quality of practical courses were: (1) unable to implement exercises, (2) poor absorption of learning content, (3) lack of practical teaching materials, (4) poor network connection, and (5) insufficient online learning resources. When more and more chances to occur online learning, people tended to trust the information gathered from the Internet, as a result, the quality of online courses could be the indicator to be determined whether students were comfortable with online courses (Lin, 2021).

**TABLE 6:** External factors affecting the learning quality of online courses.

No.	Theoretical courses		Practical courses	
1.	Environmental interference factors (such as noise)	251 (23.5%)	Unable to implement exercises	339 (31.7%)
2.	Poor network connection	228 (21.3%)	Poor learning content absorption	217 (20.3%)
3.	Poor learning content absorption	206 (19.3%)	Lack of practical teaching materials	124 (11.5%)
4.	Insufficient interaction between teachers and students	82 (7.7%)	Poor network connection	108 (10.1%)
5.	Unsmooth operation of the platform	78 (7.3%)	Insufficient online learning resources	82 (7.7%)
6.	Bad personal hardware	65 (6.1%)	Environmental interference factors (such as noise)	67 (6.3%)
7.	Others	71 (6.6%)	Insufficient interaction between teachers and students	48 (4.4%)
8.	Insufficient online learning resources	56 (5.3%)	Unsmooth of the platform usage	44 (4.1%)
9.	Insufficient interaction among students	23 (2.2%)	Others	31 (2.8%)
10.	Lack of practical teaching materials	8 (0.7%)	Bad personal hardware	12 (1.1%)

### Discussions

#### The Relationship Between Expectancy Beliefs and Course Satisfaction

From the past research, Bhattacherjee (2001b) found that expectancy performance could be an important determinant of satisfaction, because expectancy beliefs were the benchmarks that users used as reference judgments for assessing satisfaction. In addition, Chiu et al. (2005) proposed the expectation of anticipatory beliefs, allowing users to perceive the degree of satisfaction or dissatisfaction. While Thong et al. (2006) pointed out that people’s perceived level of satisfaction was determined from the initial expectancy beliefs of services and the differences between services. Bhattacherjee (2001b) also proposed that when people received services that meet or exceed expectations, there were positive satisfaction. As a result, there was an inseparable relationship between expectancy beliefs and satisfaction. When the expectancy beliefs were more positive, the better the satisfaction might be felt.

Cheng et al. (2016) proposed from the perspective of expectation value theory that student satisfaction depended on students’ expectancy beliefs prior to learning. Appleton-Knapp and Krentler (2006) also proposed that when students’ expectations were met, it was a positive predictor of satisfaction. The research of Doménech-Betoret et al. (2017) confirmed that expectancy beliefs had a positive impact on students’ perceived satisfaction. However, the verification results of this study were slightly different from previous studies. This study found that expectancy beliefs had a positive relationship with theoretical course satisfaction, but had a negative relationship with practical course satisfaction. It could be said that students with higher expectancy beliefs for online courses also improved their satisfaction with theoretical courses; on the other hand, the students perceived a lower level of practical course satisfaction if they had higher levels of expectancy beliefs.

The research of Tan and Tan (2020) also showed similar conclusions to propose that university courses could be roughly divided into two types: theoretical courses and practical courses. The theoretical courses were teacher-centered while knowledge was mainly imparted by teachers. Practical courses were student-centered while the course was mainly based on students’ hands-on practices. He (2021) pointed out that the current online courses and live courses designed by schools were only suitable for most of the theoretical courses, not for the professional and technical practical courses which were still needing the step by step guidance from the professors. Electromechanical major courses, as examples, were the courses with highly practicality and a lot of practical operations could not be totally carried out through online teaching, since communication between students and teachers was limited that hindered the teaching of practical courses (Zhang, 2020).

Most of the colleges and universities adapted online live broadcasts, online courses, and student self-learning, etc., for the theoretical courses; however, for practical courses that required hands-on operations, online teaching methods were generally still not available (Jiang et al., 2020). Chen et al. (2021) believed that the implementation effects of distance teaching varied depending on the subject area, and there were different limitations in different application level, such as clinical medicine, veterinary medicine, art, music, design, and other fields. Subject areas that required a high degree of practice, such as sports, were more difficult to explain since distance teaching restricted the presentation of the practical courses even if teachers could adequately prepare teaching materials and teaching plan.

#### The Relationship Between Course Satisfaction and Learning Effectiveness

Kujala et al. (2017) stated that when a product or service had good usability, it meant it was useful to users. ECT proposed a close relationship between satisfaction and performance confirmation (Dai et al., 2020). Alavi et al. (1995) and Li and Liang (2020) both believed that learning satisfaction was a key indicator impacting learning effectiveness. In sum, when students were satisfied with the courses, the learning effectiveness brought by the course were more positive.

Wickersham and McGee (2008) pointed out that student satisfaction was one of the important factors to be considered when developing courses, because it was directly related to whether students succeed in learning or not. Willging and Johnson (2009) also believed that learner satisfaction was an important factor affecting the effectiveness of online courses. The verification of this study showed that the satisfaction of both theoretical and practical courses had a positive relationship with learning effectiveness, as well as online courses. A higher level of course satisfaction helped to improve learners’ perception of learning effectiveness in online courses that coincided with the viewpoints in the previous literature.

#### The Relationship Between Learning Effectiveness and Continuance Learning Intention

Kujala et al. (2017) suggested that when users found out a product that helped to improve efficiency, they were more willing to continue using it. Dai et al. (2020) pointed out that when an individual’s level of affirmation about perceived performance was crucial for explaining persistence intention. Wang T. et al. (2021) suggested that ECT provided a good explanatory basis for the relationship between satisfaction and persistence intention. Hsu et al. (2018) found that for learners who had experienced in using online learning websites, the factors that affected their intention to use was the effectiveness of learning. Bai et al. (2021) more explicitly proposed that the fundamental factor affecting the effect of learning behavior on persistence intention was that the more users understood the benefits of using the tool, the more they wanted to continue using the tool. Perceived positive performance was one of the important factors affecting the user’s continued intention (Hsu et al., 2018). The verification of this study showed that the satisfaction of both theoretical and practical courses had a positive relationship with learning effectiveness. In some other words, a higher level of learning effectiveness helped to extend learners’ continuance intention to online learning.

## Conclusion and Recommendations

### Conclusion

Under the influence of the COVID-19 pandemic, China adopted the method of online learning since 2019. Through the method of suspending classes without stopping, courses were able to be continued. But for the students of vocational and technical teacher colleges, the satisfaction of learning was still relatively limited. The faculty was forced to proceed with distance teaching or other different approach to practice. Even though students accepted non-face-to-face lecturing, limitations, or near-impossible usage of hands-on equipments were still struggles to all. All the higher education sectors faced the same dilemmas (Gamage et al., 2020). Empirical studies of what was working and what was not in online learning was necessary for the public to understand (Carrillo and Flores, 2020), so students can obtain a meaningful learning experience in the online learning environment. Therefore, in this study, the structural equation model was used to verify the factors affecting the satisfaction of college students in vocational and technical normal colleges, as well as their perceived learning effectiveness and continuance learning intention in the context of online courses. The study results showed: (1) Expectancy belief had a positive relationship with theoretical course satisfaction, but has a negative relationship with practical course satisfaction; (2) Theoretical course satisfaction and practical course satisfaction had a positive relationship with learning effectiveness; (3) There was a positive relationship between learning effectiveness and continuance learning intention. When students’ expectancy beliefs were stronger, their satisfaction with practical courses was lower, but the satisfaction of practical courses was related to students’ perceived learning effectiveness for online courses and their intention to continue learning.

Doménech-Betoret et al. (2017) suggested that student satisfaction was one of the most important learning outcomes and a key indicator of educational quality. Cheng and Yuen (2018) also pointed out that users’ satisfaction always was a key factor influencing the post-acceptance behavior of users of various technologies in an organization or higher education setting. As a result, the participants had a moderately positive attitude toward satisfaction with theoretical courses, but have a negative attitude toward satisfaction with practical courses. Online practical course teachers had great room for improvement.

### Recommendations

Based on this study, students’ satisfaction with theoretical courses was relatively high in online courses, but their satisfaction with practical courses was relatively low. Therefore, it was urgent to improve the teaching form of practical courses. When students could not operate equipment and practice concurrently, it was particularly important for teachers to integrate and effectively provide alternative learning materials. Therefore, this study suggested that all professional teachers in vocational and technical normal schools should have multiple teaching strategies and develop digital teaching skills, actively design practical course materials for online-based courses to meet students’ learning needs.

Moreover, external factors affecting the quality of theoretical courses were as followings: (1) environmental interference factors (such as noise), (2) poor internet connection, (3) poor absorption of learning content, (4) insufficient interaction between teachers and students, and (5) unsmooth platform operation, which occurred a lot in people’s the daily living environment. The factors affecting the quality of practical courses were: (1) unable to implement exercises, (2) poor absorption of learning content, (3) lack of practical teaching materials, (4) poor Internet connection, and (5) insufficient online learning resources. In this study, teachers and students must overcome environmental interference factors together in class and to arrange classes in a quiet environment. They also needed to remind each other to set mute before class. For practical courses, due to the physical limitations of online courses, students could not practice, and teachers could not evaluate students’ learning effectiveness. Therefore, teachers might try to establish a compromise method of virtual practice factory. Although this method was still unable to carry out all the physical practice, students could practice procedural knowledge for some degree during the practice of virtual operation. In addition, the counseling provided by course teachers was very important for students’ learning. Regardless of whether there was a pandemic, students needed to have the opportunity to consult with teachers (George, 2020), so this study strongly suggested that teachers should provide more flexible counseling methods and schedule, so that students could have more time to overcome academic problems when they could not see the teachers in person.

During regular courses or physical face-to-face courses, each student should have their own set of books and teaching materials required for practice, so that they could listen to the teacher’s explanation and carry out practical exercises in the classroom. But during this pandemic period, students could not buy supporting textbooks, which made teaching difficult (Hu, 2020). Putra et al. (2020) found that many students expressed that online learning brought them learning difficulties and challenges. George (2020) also proposed that most students had learning difficulties at home due to insufficient learning resources, so students should first be provided with various learning resources to promote their learning in the subject area. Therefore, this study suggested that in addition to the original teaching materials, teachers should actively seek more supplementary learning materials (including videos, images, text, etc.), which could also create more opportunities for students to learn independently.

The development of vocational education is extremely important to the development of the country, society, and economy. If the development of vocational education is expected to be smooth, the quality of its teacher training is one of the key factors. However, the current discussion on teacher training in vocational education is much less than that on technician training in the vocational system; so are the number of relative researches. Therefore, the results of this study will help to increase the public’s attention to vocational teacher training education.

### Research Limitations and Future Study

This study was a cross-sectional study, conducted during the COVID-19 pandemic, so the participants were in involuntary and hasty distance learning. The future study can explore more on technical students who conduct distance learning under special circumstances such as non-epidemic conditions to discuss what is the perceived level of course satisfaction, learning effectiveness, and continuous learning intention for theoretical courses and practical courses in the normal school time. It can also compare whether there are differences in course satisfaction, learning effectiveness, and continuance learning intention brought among different online learning tools.

Studies on individual differences among students can help to understand the learning barriers that students may encounter during online learning to improve teaching methods and provide students with learning suggestions, provide more suitable teaching content, and also improve teaching methods. Table 1 represented that the sample numbers of participants in different disciplines in this study were unevenly distributed, making it difficult to compare differences. Therefore, this study suggested that participants could be recruited equally for different varieties in the future, so more information could be gathered to understand the similarities and differences of the problems encountered by vocational and technical normal students with various backgrounds, to provide more in-depth information and appropriate learning strategies.

This study confirmed that belief was an important antecedent variable affecting learning. However, this study only discussed the influence of expectancy beliefs on course satisfaction, learning effectiveness, and continuance intentions. Therefore, more influence of expectancy beliefs in online learning can be explored from more different points of views in follow-up studies. For example, it can be analyzed in the framework of the belief-action-result model, the predictive effect of expectancy beliefs on the learner’s behavioral performance, and how the learning action influences the learning effect.

## Data Availability Statement

The raw data supporting the conclusions of this article will be made available by the authors, without undue reservation.

## Ethics Statement

Ethical review and approval was not required for the study on human participants in accordance with the local legislation and institutional requirements. Written informed consent for participation was not required for this study in accordance with the national legislation and the institutional requirements.

## Author Contributions

J-HY, Y-SL, and ZH: concept and design, drafting of the manuscript, and critical revision of the manuscript. J-HY and Y-SL: acquisition of data and statistical analysis. All authors contributed to the article and approved the submitted version.

## Conflict of Interest

The authors declare that the research was conducted in the absence of any commercial or financial relationships that could be construed as a potential conflict of interest.

## Publisher’s Note

All claims expressed in this article are solely those of the authors and do not necessarily represent those of their affiliated organizations, or those of the publisher, the editors and the reviewers. Any product that may be evaluated in this article, or claim that may be made by its manufacturer, is not guaranteed or endorsed by the publisher.
